# The Paradox of Political Accountability and Deficits in the Preconditions for Service Delivery in Elderly Care: A Qualitative Study of Swedish Politicians

**DOI:** 10.3390/ijerph182312350

**Published:** 2021-11-24

**Authors:** Susann Porter, Tuija Muhonen

**Affiliations:** 1 Centre for Work Life and Evaluation Studies (CTA), Malmö University, 205 06 Malmö, Sweden; tuija.muhonen@mau.se; 2 Department of School Development and Leadership, Faculty of Education and Society, Malmö University, 205 06 Malmö, Sweden

**Keywords:** elderly care, municipality politician’s political assignment, psychosocial work environment, work environment

## Abstract

The aims of this qualitative grounded theory study were to explore how politicians accountable for Swedish elderly care viewed their assignment, their beliefs and knowledge regarding the psychosocial work environment for elderly care employees, the factors affecting their work environment, and how these politicians regarded elderly care during the COVID-19 pandemic. This study consisted of 41 interviews with politicians in municipalities across Sweden. Three categories emerged from the analysis: (1) *interpretation of the assignment directs the focus*; (2) *recognizing shortfalls in the employees’ work environment*; and (3) *exposing deficiencies due to the COVID-19 pandemic.* The strongest category was identified as *interpretation of the assignment directs the focus* and was described as the delivery of good and quality care. Nevertheless, this study highlights shortfalls in the delivery of care services where the employees’ work environment, especially in the home care sector, was frequently described as stressful. The COVID-19 pandemic adversely affected the work situation for staff in elderly care. In that setting, staff shortage and lack of competency were common. Nurses were particularly affected by high workload and responsibility. Further research should explore civil servant roles in the elderly care sector and how these actors view their collaboration with municipality politicians.

## 1. Introduction

Severe deficiencies within elderly care in Sweden became visible and were widely discussed during the COVID-19 pandemic [1,2,3]. However, deficiencies in the work environment for staff working in elderly care were reported prior to the pandemic: high sick leave rates [4,5,6,7,8] and difficulty recruiting qualified staff [8] were highlighted. Problems also extended to leadership roles. First line mangers in the elderly care sector often have many subordinate staff and can experience inadequate organizational support [9].

The Swedish health care sector consists of three levels, each governed by democratically elected politicians. These are the State (i.e., the central government), regions (i.e., the county councils), and municipalities. The role of the State is to establish principles and guidelines and set the overall political agenda for Swedish health care [10]. The 21 regions are responsible for organizing access to quality health care for all citizens including primary and hospital care [11]. There are 290 municipalities within the regions, and they are responsible for providing services including elderly care. Regions and municipalities provide direction and have decision-making power [10]. The municipality councils decide what specific areas boards and committees are accountable for, and a board can delegate an area of responsibility to a committee [11,12] Consequently, boards and committees vary among the municipalities. 

Swedish health care is regulated by the Health Care Act with the goal to provide good health care for all citizens on equal terms while prioritizing those in need [10]. The Social Services Act is an additional legislation that states that elderly care shall enable the elderly to live a dignified life and have well-being [13]. It is a goal-oriented law and does not regulate the details of care [13,14].

Within politically led organizations, primary responsibility for the work environment lies with accountable politicians because they represent the municipality as employers [15]. The Work Environment Act stipulates that employers are responsible for the physical and psychosocial work environments of their employees, and for preventing work-related illness among employees [16]. An important part of the political assignment is the setting of goals, priorities, and quality standards for the organization. A primary priority is to ensure the organization has a good work environment, and this includes high quality management with the right conditions to exercise leadership [15,16]. Additionally, staff who work in elderly care should have appropriate training and experience to execute their jobs [17]. 

Accountable politicians must ensure conditions are compliant with the relevant laws, although operational responsibility for the work environment is handled by civil servants, first line managers, and employees through their daily work [15]. Civil servants in the municipalities have the administrative role of providing the accountable politicians with an objective and impartial factual basis for making decisions. The head of administration must ensure that organization of work is conducted in accordance with the formulated goals and guidelines, and that budget frameworks are kept [18]. 

The Swedish municipalities are a major employer for elderly care staff and have approximately 275,800 employees, 239,600 of whom are women [19]. Most nursing home care is provided by care assistants, assistant nurses, and registered nurses [20], with physiotherapists [21,22], and occupational therapists responsible for rehabilitation [23,24,25]. A home care employee meets approximately 12 care recipients during a workday. This is the equivalent of about 10 residents a day in specialized care [26]. 

The relationship with the resident and the ability to provide sufficiently good care is an important aspect of the staff’s work. Feelings of inadequacy are associated with low job satisfaction and ill health [7]. Good working conditions are a prerequisite to delivering quality care for the elderly [7,27]. However, sick leave due to mental health problems is common in elderly care [5,6,28]. A Social Insurance report shows that a significant amount of sick leave is linked to work-related stress [5]. Mental health problems can occur when the demands of the job exceed employee resources more than temporarily [16]. Employees who suffer from mental health problems are more likely to have jobs with both a high level of psychological demand and low level of decision autonomy, as this combination can lead to unhealthy work-related stress [29,30]. When staff in elderly care cannot provide the care they feel is needed, it can be a significant stress factor and one of the underlying causes of mental problems [6]. 

A recent study of Swedish night-time elderly care workers showed that time was a precondition to provide quality care but was often lacking [4]. High turnover is a further problem, and several factors can impact staff consideration for leaving the sector. These factors include high workload, lack of managerial support, part-time employment, split schedules, lack of qualified staff, and lack of control [7]. Factors that can contribute to an increased intention to stay in the current workplace are linked to increased years of experience and career promotion opportunities [31].

Management plays a vital role in how elderly care staff experience the pressures of work [32]. Supportive management is associated with lower perceived work strain [33]. A systematic review showed that teamwork, employee involvement, and positive, accessible, and fair leaders were vital factors for a healthy work environment [34]. 

However, the work of managers within elderly care is largely characterized by strained finances, responsibility for many subordinate employees, and limited organizational support [9]. In 2015, there were approximately 60 employees per first line manager in elderly care and administrative support was often deficient [35]. No real changes in the number of subordinates have occurred in recent years [36], and turnover among first line managers is high. In Sweden, over 90 percent of first line managers are women. A comparison of managers in male-dominated areas shows that men on average have responsibility for significantly fewer employees [37]. Mangers in elderly care can also experience a feeling of lack of control over decisions made for their work area, and directives from accountable politicians are understood to mean that work should be performed at the same quantity and quality levels, but with fewer resources [38]. 

The COVID-19 pandemic disproportionately impacted elderly care in Sweden [1,3,39]. A quarter of long-term care facilities were affected by COVID-19 infections at the end of April 2020 [3]. While in the Swedish capital Stockholm, 67% of the long-term care facilities were affected [3]. A government report evaluating the handling of the pandemic in Sweden highlighted several deficiencies in nursing homes. These included insufficient staff competence, low accesses to nurses, and lack of access to medical equipment. The lack of nurses led to serious shortcomings in both regular care and the end-of-life care for elderly residents [1,39]. Additionally, medical assessment and hospitalization in elderly care was lacking. Some of the ill elderly did not receive adequate medical assessment, care, or hospitalization [2]. A narrative review of the work environment during the pandemic showed that health care workers were at a higher risk of developing depression, anxiety, stress, and sleep disturbance [40].

Long-term care for the elderly tends to have low political priority. Political attention to its importance is often transient and secondary to health care [3]. Consequently, the focus of this study was the accountable politicians in the municipality. The political decisions frame elderly care and all professions in the sector are directly or indirectly affected by decisions such as resource prioritization and cost savings requirements. To the best of our knowledge, no research has focused on municipality politicians’ accountability for elderly care in Sweden. Therefore, specific aims for the present study were to explore:

- how politicians accountable for elderly care view their assignment for elderly care;
- politicians’ beliefs and knowledge regarding the psychosocial work environment for elderly care employees and the factors affecting their work environment; and
- how the politicians viewed elderly care during the pandemic.

## 2. Materials and Methods

### 2.1. Study Design

The grounded theory method was selected to guide the interactive data collection and analysis process as research in the field of municipality politicians with accountability for elderly care is scarce. Grounded theory is a flexible and systematic method that enables researchers to collect and analyze substantial amounts of qualitative data describing processes and actions. A constructive approach to grounded theory was applied with the goal to construct theories from the empirical data. Grounded theory is a bottom-up approach that strengthens the method as the researchers continuously engage with the data through analytical questions [41]. The present study is part of a larger research project exploring municipality politicians’ mental health literacy and their perceptions of the knowledge needed regarding their assignment of elderly care.

### 2.2. Participants

The study inclusion criteria comprised politicians within Swedish municipalities who had been accountable for elderly care for at least one year. The included politicians were elected to be on a board or an equivalent committee in their municipality with responsibility for elderly care. One participant was included with six months accountability by fulfilling other important diversity criteria as a woman from the Swedish Democrat party. The intention was to include politicians from across geographical areas of Sweden, all political parties, genders, and both majority parties and the opposition. A broad range of political roles were included: chairs, vice and deputy vice chairs, board members, and deputy members. Additionally, the politicians had to be 18 years old or older and able to speak, read, and understand Swedish.

There were 41 study participants including 18 women and 23 men with a mean age of 61.7 years, and age range of 27–79 years. Further characteristics are shown in Table 1.

### 2.3. Data Collection

Data collection began in the autumn of 2020 and ended in the spring of 2021. The potential participants were identified from the municipality websites, contacted by email, and given information about the study. If they agreed to participate or requested further details regarding the study, this information was provided by email or phone. If politicians agreed to participate, a time was booked, and an invitation sent for a video meeting (Zoom or Teams). Verbal information was given prior to the start of the interview, and the participants had an opportunity to ask questions. Four interviews had to either be partly or fully executed over the phone due to technical difficulties. The interviews lasted between 35 and 140 min and were digitally recorded. 

Each interview started with demographic questions regarding age, academic background, years working as a politician, questions on whether they had any clinical experience working in elderly care, and a question about education in the work environment. A semi-structured interview guide with open-ended questions was developed in relation to the study aims by the authors (Appendix A). 

As suggested by Charmaz [41], the interview questions started out more general in character and were open-ended. The intention was to keep the researchers’ assumptions and theories in the background. As the interview progressed and categories emerged, more specific questions were included to test the emerging categories. Participants were encouraged to not give politically motivated answers but to try to be as factually correct and personal as possible. The interview guide was tested twice in pilot interviews, which resulted in minor changes.

### 2.4. Analysis

*Initial sampling* was applied to select the first two politicians in relation to the aims and study inclusion criteria. Interviews were transcribed verbatim and analyzed using line-by-line initial coding with the goal of locating tentative theory and to comprehend the data. *Initial coding* is part of the early process where the researchers define and label the text (e.g., coding the transcribed text with short words that reflect actions in the text). Initial coding starts to form links between the data and emerging theories and can prompt the researchers to find areas that are lacking. Initial coding is particularly useful when working with detailed data from transcribed interview material regarding fundamental empirical problems [41]. The next step is *theoretical sampling*. This involves continuing data collection through seeking to explain and elaborate tentative categories and relationships between the categories in order to create an analytical meaning. Theoretical sampling allows gradual inclusion of participants and data collection and the analysis process is performed simultaneously in an interactive process. When using theoretical sampling, the researcher can add and modify questions to explore a specific area that has developed during the analytical process. This procedure of feeding initial results back to the data collection by adding questions is a vital part of grounded theory. The theoretical sampling was considered to be concluded (i.e., theoretical saturation reached) when no new theoretical insight occurred in relation to the emergent categories. *Focus coding* was the next step. The most recurrent and important initial codes were used to categorize the data. The authors then proceeded to axial coding, which connects the categories and sub-categories to each other by giving the codes coherence to the developing analysis, relating the categories and subcategories. *Theoretical coding* was the last step and connects the categories to provide an analytical explanation. Memo writing was used during the entire analytical process to reflect on the analysis and to validate categories and codes. Memo writing was also used during and immediately after concluding the digital interviews. Negative cases (i.e., results that contrasted to the main result) were also included. Open Code version 4.03 software supported the data analysis process.

### 2.5. Ethics

The study was approved by the Swedish Ethical Review Authority (Dnr. 2020-05409). Informed consent was obtained from all participants, including consent for digital recording. All participants were guaranteed confidentiality and the right to end their participation without giving a reason. All procedures involved in the research were performed in accordance with the ethical guidelines of the Declaration of Helsinki regarding research on humans [42]. The audio files and all the analytical material are stored in a secure area at Malmö University with access only to the researchers involved in the study. All researchers for this study are employed at Malmö University. The transcribed material does not include participant names, locations, workplace, or other information that could be linked to the participant. The first author (S.P.) who conducted all of the interviews is experienced in interviewing and executing qualitative studies.

## 3. Results

Emails with information about the study and a request to participate were sent to 456 municipality politicians who met the inclusion criteria, across the 290 municipalities in Sweden. Of those 456, 401 (88%) did not reply whilst 14 (3%) replied and declined to participate (Appendix B). A total of 41 (9%) politicians chose to participate (Table 1). Diversity across gender, political affiliation, and governing mode (i.e., in majority and opposition mode) was sought (Appendix C). Most of the politicians who participated (37 out of 41) were also members of a board with accountability for other health care areas besides elderly care. These areas included substance abuse treatment, and children, young people, and adults who need care and support. Of the politicians who participated, four worked full time as politicians and 37 worked part-time. 

Three main categories (Table 2) related to the study aims emerged. The strongest category was the first: interpretation of the assignment directs the focus, recognizing shortfalls in the employees’ work environment, and exposing deficiencies due to the COVID-19 pandemic.

The politicians regularly referred to themselves as employers for the staff in elderly care. The civil servants were referred to by the politicians in their various roles (e.g., the head of administration, the head of operations, and the head of social services). In the present study, the term civil servant is used to refer to all these different roles. The first line managers of staff engaged in elderly care were not included within the definition of a civil servant in this study. In the results, references to nursing homes encompassed the various forms of accommodation provided by the municipality where the elderly receive support from professional staff. In the quotations provided in the results section, the politicians’ governing mode is shown to be either in the majority or opposition. Politicians in the majority included both governing the municipality with their own party majority or in coalition with other political parties. In Sweden, chair positions come from the majority party.

### 3.1. Interpretation of the Assignment Directs the Focus

This category represents the politicians’ understanding of their political assignment toward care of the elderly. Some of the politicians provided extensive explanations of what they believed their assignment consisted of and provided examples to show their interpretation. Others gave a short response using a few sentences. When asked to define the assignment, many paused before answering. Occasionally, politicians qualified their definitions by stating that this was how they interpreted the assignment, leaving it open for others to view the assignment differently.

#### 3.1.1. Delivering Good Care When Needed

Overall, the politicians were clear that their assignment was to be responsible for elderly care in their municipality including those who lived at home and those who lived in a nursing home. Some specified that the assignment was to ensure that the elderly received good care when they needed it, that they were able to live independently in as fulfilling a life as possible, and to strengthen health among the elderly. A common view was that quality care should be given, although good care and quality were not specifically defined. A second vice-chair from the Social Democrat party in opposition explained her view on the assignment:

“*I have always viewed it to be my assignment to deliver as good care as possible for those who need care and nursing … We must always make sure that we deliver the best there is. The best depends on what one thinks is the best based on what kind of values one has.*”

Participant 24

Some stated that their assignment was to fulfil the legal obligations within the Social Services Act, and to ensure that civil servants followed the law when proposing reorganizations or budget cuts. A female, board member from the Environment party in opposition explained:

“*It becomes a bit of an ideological issue, but somewhere it is to do the minimum. We must at least do what the law requires….*”

Participant 28

A couple of participants stated that the politicians’ understanding of their duty was in conflict because most of the politicians on the board did not know the laws that regulate elderly care. A male committee member from the Center Party in opposition explained:

“*We [politicians] should monitor the decisions in accordance with laws and the legal requirements that affect the area [elderly care], and there you can say that it feels a bit comical because none of the politicians who sit on the board have even looked in the law book and seen if the proposal that the civil servants invoke is correct. It’s a bit comical at the same time as it is very serious.*”

Participant 18

#### 3.1.2. Setting Goals and Directions 

Another common description of the political assignment was to set goals and directions for elderly care in the municipality, to lead and move the board forward, and to develop elderly care. Some of the politicians stated that their role as politicians was to be the citizens’ representative toward civil servants. Furthermore, the word ‘vision’ was also used to describe the political assignment of elderly care. As a male chair from the Social Democrat party explained:

“*We are responsible for these overall big issues; what are the focus goals, where are we going, what are the visions for nursing […].*”

Participant 7

Most of the politicians stated that their job was not to manage detailed decisions in the organization because that was the civil servants’ and first line managers’ responsibility. The politicians’ role was to set the goals and direction for what the municipality wanted to achieve. How to reach those goals was then for the civil servants and first line managers to determine. A board vice-chair from the Social Democrat party in majority described how he viewed the political assignment:

“*Politicians must paint the big brush strokes when it comes to elderly care ….*”

Participant 2

It was considered as a dilemma to either be too involved in operations regarding the organization of elderly care or not to be involved enough. A board member from the Center party in opposition elaborated on how he thought of this dilemma:

“*If you put it simply, what we as politicians are responsible for is to set goals: the focus, scope, and quality. The actual leading, the concrete measures to meet politicians’ expectations, it’s the civil servants’ responsibility. It’s difficult to draw boundaries there. Sometimes I think you get too involved and sometimes not involved enough.*”

Participant 3

#### 3.1.3. Being Responsible for the Employees’ Work Environment

This sub-category included the politicians’ views and beliefs about their role as employers with a responsibility for the work environment for staff working in elderly care. Whilst the politicians referred to themselves as employers for the staff, only a few mentioned unprompted that the political assignment included accountability for both the outcome of care delivery to the elderly, and the staff delivering the service. Nevertheless, when asked directly, most politicians believed they had, or could be considered to have, responsibility for the work environment and to make sure the environment was good for staff working in elderly care. As a male board chair from the Moderate party stated:

“*We as politicians have absolute employer responsibility. This is an issue that has been discussed a lot. It’s a politically controlled organization, not a civil servants’ organization. That is very important to know.*”

Participant 34

Some politicians elaborated further on their responsibilities when staff became ill due to their work environment, and their responsibility to provide the administration with the right conditions to prevent the staff from becoming ill. There was concern from these politicians that they did not fulfil that responsibility. Some stated that whilst they were responsible for the work environment, this responsibility was delegated either to the civil servants or first line managers. Whilst responsibility was delegated, some politicians believed that they were still accountable with a requirement to follow-up on the delegation as a woman board member from the Social Democratic party in opposition elaborated:

“*Ultimately it’s politicians who are responsible for the work environment…. We delegate that task, but that is based on that we follow up that delegation…. We have a work environment responsibility for the staff even if we delegate it to the first line managers.*”

Participant 20

The politicians considered themselves as employers with an obligation to know the laws that regulated elderly care, although the requirements of the Work Environment Act were rarely known. The civil servants were often thought of as having direct responsibility for both the work environment and the first line managers. There were politicians who felt less accountable and stated that they as politicians had limited responsibility for the work environment as that responsibility was delegated to the civil servants.

### 3.2. Recognizing Shortfalls in the Employee Work Environment

This category represents the understanding and views on the level of sick leave among staff, work conditions, and the work environment specified by the interviewer as occurring in the time prior to the COVID-19 pandemic. Various views of the politician’s own ability to affect the employees’ work environment were stated.

#### 3.2.1. Sick Leave Being a Major Problem 

Sick leave among the staff in elderly care was considered a major problem that concerned the politicians. Nevertheless, some politicians lacked awareness of the sick leave rate, and most did not know why staff were on sick leave. While a few politicians stated that the sick leave rate was not very high, the majority viewed sick leave as a major problem within elderly care. About half of the politicians reported sick leave rates among staff in elderly care ranging from 6% to 30%. A male board chair from the Christian Democrats expressed:

“*We have an average of 10% sick leave and I felt when I came in [to the board] that cannot be acceptable. I cannot run an organization where every tenth employee is not at work—then something is wrong. We [the board] have a work environment responsibility and should make sure the staff are well.*”

Participant 12

The politicians received regular board meeting presentations from the civil servants including the sickness absence rate, but the root causes were typically not given. Instead, sick leave was often described as either short- or long-term sick leave and not divided into physical illnesses or mental health problems. A couple of the politicians had questioned the civil servants as to why staff were on sick leave but had been assured that this was due to the employees’ private lives or serious (non-work related) illnesses rather than the work environment. A committee vice chair from the Moderate party in majority expressed her views regarding sick leave:

“*The sick leave rate was at 30% a few years ago in a team, which was completely horrendous. The explanation we got [from the civil servants] when discussing the sick leave rate was that it was stomach ailments or the flu, but, I mean, that comes around every year, so that is a poor explanation.*”

Participant 14

A board member from the Swedish Democrat party in opposition elaborated that the sick leave rate had started to decrease, as he described:

“*It’s between 15 and 20 percent I think, it has gotten better.*”

Participant 27

Some politicians believed that there were staff in elderly care who struggled to work full time. The politicians suggested that these individuals should be able to work part-time if that was better for their health. Some of the politicians blamed a dysfunctional culture in certain teams where employees who did not get what they wanted at work declared themselves sick. A few politicians even suggested that the work was not considered important by some staff in elderly care, who were therefore more often on sick leave while not necessarily ill. Additional explanations included a lack of good leadership in elderly care that could result in staff sick leave. A board chairwoman from the Christian Democrat party said, regarding the high sick leave rate among care assistants in home care:

“*There is a high level of sick leave in home care, but I don’t know why that is. It could be that they are not happy with their boss. It could be that they are very ill. I don’t know, but there is a high level of sick leave and I cannot believe that it’s due to the work environment.*”

Participant 17

A few politicians had been informed by civil servants that the cause of sick leave was mental health problems. The work environment may be a factor behind sick leave due to mental health problems because working in home care was described as stressful. Nearly all politicians believed that the reasons for the high levels of sick leave were important information to have, although the majority had never asked questions on the topic. Some said during the interviews that this needed further questioning and resolved that they would enquire about it at the next board meeting.

#### 3.2.2. Lacking the Prerequisites for Work

This sub-category represents the politicians’ view of the employees’ working conditions in elderly care. Several aspects were identified as negatively affecting the work environment. The politicians’ opinions regarding the employee work environment was based on meetings with staff during nursing home visits or with home care teams, direct contact by staff via phone and email, or a personal approach when out in their community. These views could also be based on civil servant presentations or first line manager perceptions of the work environment. Especially in smaller municipalities, politicians often had informal contacts through friends or a direct relationship with someone who worked in an organization.

The collective view was that stress was common and negatively affected the work environment and ability to perform the work. While stress among nursing home staff existed, the problem was often perceived to be worse among the staff in the home care sector. These employees often worked alone, and this was compounded by travel distances between residences and a tight time schedule. Within home care, the minute-by-minute schedule was brought up as a common way of working; different tasks were assessed to take a certain standardized time that was based on the elderly person’s needs as determined through a support assessment decision. Overall, it was more difficult to obtain trained staff to work in home care. The specific time schedule was often viewed as a negative factor for the work environment that led to stress and a feeling of not doing a good job because time was limited and measured in minutes. Additionally, a female board vice-chair from the Moderate party in opposition commented about the timed schedule:

“*There can be very ill individuals who need a lot of help, and how do you calculate that? I have asked it, tell me how to calculate a shower aid? They (elderly) should undress, shower, and have help with this in 15 min….*”

Participant 9

When staff were absent on sick leave, it could be difficult to find temporary workers to substitute. Uncertainty about sick leave coverage could cause stress. Available temporary workers were often unqualified and might struggle with the Swedish language. A woman from the Liberal party board member in opposition elaborated on the lack of qualified staff:

“*There is a shortage of staff. There are no people to get a hold of, for example, during the summer…. I got the comment [from a staff] that you cannot hire a person just because they have two arms and two legs. They must understand what you are saying, too.*”

Participant 41

This participant elaborated further that half of the nurses originated from outside Sweden and that without those nurses, elderly care would not function. Statements were made that it was extremely difficult to hire qualified staff and that employers had to completely train new staff once they been hired. Assistant nurses were often sought for permanent positions, but that qualification was seldom available. Employing qualified staff in nursing homes was easier than for home care. The home care sector had a higher number of sick residents who needed more care, and did not work well when the staff were less well-trained and had limited experience with very ill clients. Some said that staff might therefore make mistakes, and if the mistakes were severe, they were presented to the board as deviation reports.

The politicians considered social contact (e.g., taking time to sit and talk with the elderly) as an activity that residents needed but that staff found difficult to allow time for. Concerns were raised about paying for social contact, although it was recognized as a problem that the elderly could be isolated in today’s society. The work schedule also contributed to a poor work environment because of split shifts, work shift duty every other weekend, and long shifts with limited time for recovery. A board member in opposition from the Left party expressed her opinion about why people did not want to work in elderly care:

“*Who wants to work with elderly care when you know that you have every other weekend, and shared shifts, low pay and a stressful job that makes you sick? Yep, I want that job! Probably not!*”

Participant 31

Some politicians said that the work environment in their municipality was good without high rates of sick leave. Two municipalities worked with a Salutogenic perspective and a few had nursing homes operated by employee-owned entrepreneurs. These approaches were perceived as giving staff more influence over their work and considered to be a positive factor for the work environment that led to a decrease in sick leave prevalence. 

Annual employee surveys were used as a measure of how staff perceived their work environment. Some politicians thought the surveys were a good measure, while others thought the survey questions were formulated so that answers were difficult to decipher. Some politicians were resigned to the fact that a lack of qualified staff was just the way it was, while understanding that those in need of services and their relatives were not content. A male board chair from the Christian Democrat party expressed:

“*If staff who are completely uneducated come home to my mother, it’s clear I’m not happy. But if we do not have anyone else (to work) that day, what do we do then? It’s perhaps better that a person comes even if they are completely uneducated than nobody at all.*”

Participant 12

Very few of the politicians stated that they had the ability to influence and affect the work environment for staff either way.

#### 3.2.3. Excessive Workload and Inadequate Support to Managers 

First line managers were believed to have an important role in the organization of elderly care because of direct contact with the staff. Concerns were raised that first line managers had a tough and fragmented job, and had to deal with many employee issues, elderly in nursing homes, elderly in home care, relatives of the elderly while also keeping to a budget and executing decisions made by politicians. 

First line managers were often young, newly graduated women. Politicians were aware that support to these first line managers could be lacking and that there was high turnover. A woman from the Social Democrat party and board member in opposition conveyed the following about the first line managers’ responsibility:

“*They have a large group of employees, and then they also have to deal with all the substitutes and hourly paid employees, which can be almost twice as many….*”

Participant 20

The politicians were aware that the workload for first line managers was high, and responsibility for 40 to 50 staff was common. Some politicians stated that first line managers also had direct work environment responsibility even though they were often seen as lacking knowledge in the field and needed more resources to manage issues related to the work environment. The role of a first line manager was considered a tough job and managers often did not stay long in that position. One explanation for the high turnover was that first line managers were often starting families, and the job was too demanding for their life situation. 

Some first line mangers were open about their stressful work situation, but the majority remained silent, did not complain, and tried to maintain a positive attitude. The politicians were aware that the information provided to them by these mangers might be filtered. Some explained that when the first line managers made presentations to the board, their supervisors often checked what they were going to say. This was perceived by the politicians to be filtering the presented information.

### 3.3. Exposing Deficiencies Due to the COVID-19 Pandemic 

This category describes how municipality politicians viewed the effects of the COVID-19 pandemic on elderly care in their municipalities. Views were divided about how their own municipality managed, although none of the politicians took direct responsibility for the elderly who died of COVID-19 or the pandemic impacts on staff.

#### 3.3.1. Deficient Work Environment during the COVID-19 Pandemic 

The politicians acknowledged that during the pandemic, the work environment in elderly care changed in several ways for both the elderly and staff. New rules were implemented, and staff were required to stay at home if they displayed any symptoms of an infection. Nursing homes were closed for visitors and medical doctors were advised to avoid nursing home visits. The high number of staff on sick leave led to a shortage of not only qualified, but all staff, and a difficult work situation resulted for those still working. A male board member from the Social Democrat party in majority explained:

“*We have had a nursing home, a large nursing home, where we have been hit hard [by COVID-19], and not just among the elderly residents. The big problem now is the lack of staff. The staff are sick.*”

Participant 11

Politicians accountable for elderly care were aware that staff had been concerned with shortages of personal protective equipment, particularly at the start of the pandemic in the first quarter of 2020. Even though most politicians had nursing homes in their municipality in which the elderly had become infected and died of COVID-19, several of the politicians reflected that they had either done well, or coped with the pandemic relatively well, and that the staff had acquired sufficient competence to care for the elderly when severely ill from COVID-19. A board chairwoman from the Social Democrat party did not believe that elderly care in her municipality had been inadequate even though many elderly residents in care homes had died from COVID-19. She explained:

“*Many have died, tragically…. Now we have examined the whole process, and what we have done and what we have not done, medical equipment and knowledge and everything, we can’t see that what happened was due to shortages of equipment, that they [staff] did not follow routines, that there was a lack of knowledge, that there was no protective equipment. We cannot see that these were the reasons.*”

Participant 19

Opposing views were also expressed that the elderly had been hit hard by the pandemic, the staff had to be infection specialists to cope, and that the demands on staff had been extremely high. A committee vice chair from the Moderate party in majority stated how she felt her municipality had coped:

“*Of course, many of the older, fragile people have been sacrificed, and that feels very difficult.*”

Participant 14

Temporary workers were often used to cover the staff on sick leave, and they could lack the skills to care for the elderly. A male board member from the Social Democrat party in majority explained:

“*… those who did not have an education did not have a clue of even basic hygiene routines…*”

Participant 26

#### 3.3.2. Considering the Substantial Staff Responsibility

The politicians explained that the municipalities did not employ medical doctors. Instead, medical doctors were hired by the regions, and were often stationed at primary care health centers. The regional authority had instructed doctors not to visit nursing homes due to the infection risk, and instead, assessment was usually performed via phone with the responsible nurse. The elderly who were ill were often treated at nursing homes by available staff. Views were divided as to whether phone assessment was acceptable or not. Some said that phone assessments were used only when the doctor already knew the patient and therefore were acceptable; the contrary view was also stated (i.e., that a phone consultation for a seriously ill patient was unacceptable and not all doctors knew the elderly residents they assessed). A committee vice chairwoman from the Moderate party in majority explained:

“*Of course, I belong to those who think that the municipality should hire its own doctors to deliver the whole chain [of health care] and corona has shown that this is what is needed. Of course, it is not possible to assess all patients on the phone. It’s not possible.*”

Participant 14

Nurses were viewed as having a leading role because they had the highest medical competence and were thought of as having a tough work situation under normal circumstances. During the pandemic, the nurses’ jobs became particularly tough and stressful as they were the profession with medical competency and often worked alone. The nurses had to delegate duties to the other staff to cope with their workload. A board member from the Social Democrats in majority explained her views:

“*Nurses often do duties that would be performed by a specialist in a hospital.*”

Participant 37

Some of the politicians explained that they had not known that the elderly in nursing homes had not received necessary health care during the COVID-19 pandemic. 

## 4. Discussion

This qualitative grounded theory study aimed to explore how politicians accountable for the Swedish elderly care viewed their assignment, the work environment of the elderly care employees, and how these politicians regarded elderly care during the COVID-19 pandemic. The strongest finding showed that interpretation of their political assignment might direct the politicians’ focus. The assignment was stated as responsibility for the care of the elderly; those living at home and supported by care givers; and those in nursing homes. This response is in accordance with the applicable Swedish regulation, The Social Service Act [13]. The political assignment was also said to include setting goals and directions for elderly care. Delivery of the care was stated to be good and executed with quality, although neither good nor quality were specifically defined in this context. The emphasis of elderly care in the Social Service Act is on the elderly living a dignified life and with well-being [13]. The law does not define the meanings of dignified or well-being, which leaves the interpretation up to the accountable politicians.

When describing their assignment, the politicians rarely stated that they were also accountable for the employees delivering elderly care and their work environment unless prompted. In Sweden, the Work Environment Act regulates the employers’ far-reaching responsibility for their employees’ physical and psychosocial work environments [15,16]. Whilst the politicians referred to themselves as employers and were concerned about the extent of sick leave amongst elderly care staff, they often lacked knowledge of the causes of sick leave (i.e., physical or mental health problems). According to the Work Environment Act, the employer is responsible for taking all necessary measures to prevent employees from becoming ill due to their work [16]. To take on that responsibility, the authors argued that simply knowing if sick leave is short- or long-term in duration, as shown in the present study, is not sufficient information for fulfilling that responsibility. To prevent sick leave due to the work environment, analyzing the root causes of the sick leave, is of great importance. Undoubtedly, having sick leave to a degree as these results showed (6–30%), along with recruitment problems and high turnover, should be considered as a major concern for any employer.

Interestingly, the politicians had extensive knowledge of the work prerequisites in the elderly care sector, and that it could be experienced as negative where stress among the staff, especially in-home care, was common [4]. They gave examples of the lack of prerequisites for a healthy work environment (e.g., the minute-measured task schedule, split shifts, lack of qualified staff, heavy workload, and lack of time for staff to converse with the elderly). However, the politicians rarely mentioned a causal connection between the lack of good and prerequisites in the work environment, sick leave for mental health problems, and the lack of quality care delivery for the elderly. The authors argued that this illustrates a paradox in the politicians’ view of their assignment of providing good and quality care to the elderly, and the simultaneous lack of knowledge of how inadequate prerequisites can negatively affect the staff’s mental health and therefore the quality of care delivered.

Previous research has shown that mental health problems are common among elderly care staff [5,27,43,44], and that a link exists between the quality of care and the work environment [4,45]. As the lack of prerequisites was well known to the politicians, one can ask why these politicians did not place greater focus on changing work conditions and gaining a deeper understanding of the causes and solutions for the high sickness absence rates. Without greater knowledge, understanding, and action, the same problems of high sick leave rates among staff in elderly care will likely continue year after year [7].

Several explanations may exist for this lack of attention to work environment issues from the accountable politicians. Whilst knowledge of the employees’ deficient prerequisites was known and regular information on the high sick leave from the civil servants was provided, there is unclear responsibility when staff become ill due to their work. This may be why there is less focus on improving the work environment. Other possible explanations could be the delegation of that responsibility to civil servants and first line managers, the potentially short period of service time served as a political board member compared to time required to implement enduring solutions, and the broad range of other political duties. That said, ultimate responsibility for the work environment cannot be delegated and always lies with the employer [11,15]. 

Encouragingly, there were nevertheless some politicians who saw the connection between the lack of prerequisites and mental health problems. A few even stated that as politicians, they did not fulfil their obligation as employers according to the Work Environment Act [15,16]. Information provided to the politicians regarding sick leave and other issues in elderly care were mainly delivered by civil servants and first line managers. Notably, politicians were aware that they may be given filtered information that portrayed a more positive picture of elderly care than that of the reality. The employee survey was not thought of as always measuring the work environment in an accurate way, and the results were difficult to interpret. One could ask why politicians were not provided with comprehensive and accurate information for their areas of accountability. There could be a variety of explanations that should be studied further. 

When describing the impacts of the COVID-19 pandemic on elderly care, politicians said they were given regular updates by civil servants. The general belief was that their own municipality coped well during the pandemic, even though most municipalities in this study had elderly nursing home residents who had suffered and died with COVID-19. The competence described as essential by the politicians was mainly training in hygiene techniques [46]. This is in contrast to the background of also describing a shortage of qualified staff due to the high rate of sick leave. Having training primarily in hygiene techniques was undoubtedly insufficient knowledge to care for severely ill and dying elderly residents. Indeed, a Swedish Government report [26] described how elderly care staff lacked qualifications, inadequate medical assessment, and a reluctance to hospitalize residents suffering from COVID-19 all negatively affected sick elderly residents [26]. Against the background of the lack of prerequisites and qualification among staff prior to the pandemic, it is surprising the politicians were not more concerned with the quality of elderly care and the employees’ working conditions during such an extraordinarily severe situation. One wonders whether the politicians were fully informed since they were not able to visit nursing homes and talk directly to the employees. Previous research has identified challenges in the collaboration between politicians and civil servants. Politicians can perceive that civil servants may withhold important information [47,48]. Whether this was an issue during the COVID-19 pandemic should be studied further. 

### 4.1. Implications for Practice and Research 

Consideration of both the elderly who need services and the employees’ delivering care is vital for politicians with the obligation and power to set the goals and directions for elderly care. A prerequisite for high quality elderly care is that staff have a healthy work environment [4,31,45,49]. Defining the term ‘quality’ in relation to elderly care is important as that would help politicians, staff, and first line managers work more actively toward a defined goal and lowers the risk of subjective interpretations. Improved prerequisites could decrease levels of sick leave among the staff and reduce the high turnover of first line mangers, consequently, increasing the quality of the care delivered to the elderly. Additionally, it is important to be clear on the delegation of work environment responsibility, so that responsibility for assessing and working actively and continuously to improve the work environment for all staff including the first line managers, is clearly owned. The first line mangers are in direct contact with the staff and are therefore crucial in the work toward a positive work environment.

The politicians in this study showed extensive awareness of the deficits in the employees’ work environment and therefore possess important knowledge of what needs to change to improve the prerequisites for the staff. A few of the politicians had changed their work method by increasing the influence of the staff in their own work (Salutogenic method and working with staff owned care homes). The politicians believed that these actions decreased employee sick leave rates in their municipalities.

### 4.2. Strength and Limitations

This grounded theory study follows the guidelines established by Charmaz including the application of criteria to increase trustworthiness [41]. The four criteria are: *credibility, originality*, *resonance*, and *usefulness*. The *credibility* criterium means that researchers achieve familiarity with the topic during the data collection and the analysis process. When planning this project, feedback was provided from two research groups within Malmö University that had extensive experience with qualitative methods. Theoretical sampling allowed for the gradual inclusion of politicians to elaborate and refine categories and to explore connections between them. The categories, sub-categories, and codes were systematically compared throughout the entire analysis process. Memo-writing was used continuously during the analysis, and during and immediately after concluding the digital channel interviews. The results were presented to two research groups at Malmö University when theoretical saturation was considered reached, and feedback was collected. 

A study limitation is that only 41 of 456 politicians contacted agreed to participate, and those had a relatively high mean age. The study would have benefited from including direct observations on the politicians at work as an additional data source. However, this was not possible since the data collection was conducted during the COVID-19 pandemic. *Originality*, the second criteria, was reached to the best of the authors knowledge as no study has been conducted with the same aims, and research in this field is lacking. R*esonance* reflects on whether the grounded theory results apply to other individuals who share the same circumstances as the included participants. The present study included a diverse group of politicians (different genders, all the major Swedish political parties, different assignments on the board or commitments) to obtain broad insight into the views of municipality politicians regarding the study aims. While one could argue that study participants were too diverse to draw strong conclusions, the aim with grounded theory is not to generalize but to develop a conceptual model that clarifies the result through identify connections and possible explanations between the categories and subcategories [41]. Two politicians who were not participants in the study had the opportunity to provide feedback on the results. However, as the comments were in line with the authors’ interpretations, no changes were made. The study included negative cases with the purpose to present a broad collection of opinions. Quotations were used to furnish a deeper sense of the participants’ perspectives and to provide transparency. The final criterion, *usefulness*, was satisfactory as Swedish politicians were responsible for elderly care, there were high sick leave rates among staff working in the sector, and problems with recruiting and retaining staff. 

### 4.3. Further Research

Further research should explore the civil servant’s role in elderly care and how these critical actors view their collaboration with municipality politicians.

## 5. Conclusions

This study contributes to a deeper understanding of elderly care in Sweden from a new perspective, that of the accountable politicians. It is important that these politicians have a clear understanding of their assignment and of their responsibility as employers of the staff working within elderly care. There are well known and inadequate prerequisites in the work environment, but the link between this unsatisfactory work environment, high sick leave rates, and the quality of elderly care has not always been acknowledged by accountable politicians. This coexistence of accountability for delivering good and quality elderly care and awareness of fundamental deficiencies that impact the delivery of care highlights a fundamental paradox where the staff and the elderly are caught in the middle.

The politicians’ views of the impacts of the COVID-19 pandemic on elderly care were generally that their own municipality had coped well. This was despite knowledge of the persistent problems that existed before the pandemic that were exacerbated by the challenges dealing with the COVID-19 pandemic. There were inadequate numbers of qualified staff, and deficiencies in the work environment exacerbated by high sick leave rates and recruitment shortages. To make elderly care a more attractive sector in which to work, accountable politicians should fully consider and act on their responsibility as employers to break the cycle of high sick leave rates, staff turnover, and recruitment problems. All staff deserve the right prerequisites for their work to be able to deliver good and quality care to the elderly while having a healthy and satisfying work life.

## Figures and Tables

**Table 1:** Characteristics of the study participants (*N* = 41).

Characteristics		*n*	Female/Male
Sex			18/23
Mean age in years (range)	61.7 (27–79)		
Education level in years	Upper secondary > 16	8	
	University > 18	33	
Professions *n* = 51 ^1^	Nurse/Midwife	6/2	
	Physiotherapist/Medical doctor	1/1	
	Nursing assistant	3	
	Social Worker/Psychologist	2/1	
	Engineer/Finance	3/6/	
	Legal/Mathematician/ Statistician	1/1/1	
	Police/Military	1/1	
	Teacher/Headteacher	1/3	
	Special educator/Caseworker Social insurance agency	3/1	
	Fire/Environmental inspector	1/1	
	Political science/Health science	1/1	
	Humanities/Agriculture	1/1	
High school education ^2^	Technology/Social science/Natural sciences	5/1/2	
Political assignment with responsibility for the care of the elderly	Board Chair	13	3/10
	Board Vice & Deputy Vice Chair	6	5/1
	Board member	17	8/9
	Deputy board member	1	1/0
	Committee Chair	1	0/1
	Committee Vice Chair	1	1/0
	Committee member	2	0/2
Mean years politically engaged (range)		17.3 (2–55)	
Mean years responsible for elderly care (range)		6.8 (0.6–25)	
Own experience of working with elderly individuals (yes/no)		21/20	
Education in work environment (yes/no)		30/11	

^1^ Participants could state one or more professions or educations. ^2^ Profession not specified.

**Table 2:** Politicians’ views of their assignment and the employees’ work environment.

Categories	Sub-Categories
Interpretation of the assignment directs the focus	Delivering good care when needed. Setting goals and directions. Being responsible for the employees’ work environment.
Recognizing shortfalls in the employees’ work environment	Sick leave being a major problem. Lacking the prerequisites for the work. Excessive workload and inadequate support to managers.
Exposing deficiencies due to the COVID-19 pandemic	Deficient work environment during the COVID-19 pandemic. Considering the substantial staff responsibility.

## Data Availability

Given the restrictions from the Ethical Review Board and that sensitive personal data were handled, the data are not freely available.

## Appendix A. Interview Questions

- How would you describe the assignment you have as a politician for the care of the elderly?
- Can you describe who is responsible for the staff’s work environment?
- How would you describe the psychosocial work environment for staff in elderly care?
- How do you view the staff work conditions for carrying out their work?
- How would you view the staff qualifications to carry out their jobs?
- How do you feel about sick leave in home services and nursing homes?
- Do you know why staff are on sick leave, if it is due to physical or mental health problems?
- How can you, as a politician responsible for elderly care, affect the staff’s work environment?
- Can you describe your collaboration with the first line managers in nursing? Is there a support function for them?
- Have you received signals that there is a lack of resources in elderly care?
- Have you, in the role as a politician, visited a nursing home and met the staff at their workplace? If so, what do the staff tell you?
- In your view, how has your municipality handled the pandemic?
- How have the staff coped during the pandemic?
- Have the staff had the right training to care for the ill elderly suffering from COVID-19?
- How have the nurses coped during the pandemic?
- What is your view of doctors not visiting nursing homes, but instead receiving a handover by the responsible nurse?

## Appendix B

**Table A1:** Dropout analysis of the municipality politicians contacted for this study (*N* = 456).

Characteristics	*N*
Politicians contacted via email (between 1 and 4 in each municipality)	456
No reply	401
Replied and accepted	41
Replied and declined to participate Women/men	14 12/2
Replied and declined, and stated a reason for not to participating Women/men	8 6/2
High workload	2
Personal reasons	2
New at the job	1
On paternity leave	2
Not suitable due to too much knowledge in the field	1

## Appendix C

**Table A2:** Political party and governing mode of the study participants (*N* = 41).

Political Party	*n*	Female/Male	Governing Mode in the Municipality	*n*
The Social Democrats	13	7/8	Majority/Minority Government Coalition/Opposition	3/2 6/2
The Moderates	8	3/5	Majority/Minority Government Coalition/Opposition	3/1 2/2
The Centre Party	4	0/4	Coalition/Opposition	2/2
The Christian Democrats	3	2/1	Coalition	3
The Liberals	3	2/1	Coalition/Opposition	1/2
The Swedish Democrats	3	2/1	Opposition	3
The Green Party	3	3/0	Opposition	3
The Left Party	2	1/1	Opposition	2
Local Independents	2	1/1	Coalition	2

## References

[1] The Health and Social Care Inspectorate (2020). No Regions Have Taken Full Responsibility for Care and Treatment (Ingen Region har Tagit Sitt Fulla Ansvar för Individuell vård och Behandling). https://www.ivo.se/publicerat-material/nyheter/2020/ingen-region-har-tagit-fullt-ansvar-for-individuell-vard/?.

[2] Szebehely M. (2020). The Impact of COVID-19 on Long-Term Care in Sweden. https://ltccovid.org/wp-content/uploads/2020/07/The-COVID-19-Long-Term-Care-situation-in-Sweden-22-July-2020-1.pdf.

[3] World Health Organization (WHO) (2020). Preventing and Managing COVID-19 across Long-Term Care Services: Web Annex. https://www.who.int/publications/i/item/WHO-2019-nCoV-Policy_Brief-Long-term_Care-2020.1.

[4] Andersson K., Sjölund M. (2020). Swedish eldercare within home care services at night-time: Perceptions and expressions of ‘good care ‘from the perspective of care workers and care unit managers. Nord. Soc. Work Res..

[5] Social Insurance Agency (Försäkringskassan). Sick Leave Due to Mental Disorders. (Sjukfrånvaro i Psykiatriska Diagnoser. https://www.forsakringskassan.se/wps/wcm/connect/e12b777c-e98a-488d-998f-501e621f4714/sjukfranvaro-i-psykiatriska-diagnoser-socialforsakringsrapport-2020-8.pdf?MOD=AJPERES&CVID=.

[6] Swedish Association of Local Authorities and Regions (SKR) (2017). Sick Leave in Municipalities and County Councils—What Is the Problem? (Sjukfrånvaro i Kommuner och Landsting—Vad är Problemet?). https://skr.se/skr/tjanster/rapporterochskrifter/publikationer/sjukfranvaroikommunerochlandsting.27519.html.

[7] Szebehely M., Stranz A., Strandell R. (2017). Who Will Work in the Elderly Care in the Future? Working Paper/Department of Social Work. (Vem ska arbeta i framtidens äldreomsorg?). https://www.socarb.su.se/polopoly_fs/1.320035.1486983623!/menu/standard/file/Slutversion%20rapport%20feb13.pdf.

[8] Harrington C., Choiniere J., Goldmann M., Jacobsen F.F., Lloyd L., McGregor M., Stamatopoulos V., Szebehely M. (2012). Nursing home staffing standards and staffing levels in six countries. J. Nurs. Scholarsh..

[9] Hagerman H., Engström M., Wadensten B., Skytt B. (2019). How do first-line managers in elderly care experience their work situation from a structural and psychological empowerment perspective? An interview study. J. Nurs. Manag..

[10] Swedish Government (Sveriges Riksdag). The Health and Medical Service Act. (Hälso och Sjukvårdslagen). https://www.riksdagen.se/sv/dokument-lagar/dokument/svensk-forfattningssamling/halso--och-sjukvardslag_sfs-2017-30).

[11] Municipality Act (Kommunallagen). https://doi.org/2017725_sfs-2017-725.

[12] Political Organization in Municipalities and Regions (Politisk Organisation i Kommuner och Regioner). https://webbutik.skr.se/sv/artiklar/politisk-organisation-i-kommuner-och-regioner.html.

[13] Social Service Act (Socialtjänstlagen Socialtjänstlagen). https://www.riksdagen.se/sv/dokument-lagar/dokument/svensk-forfattningssamling/socialtjanstlag-2001453_sfs-2001-453.

[14] Szebehely M., Trydegård G.B. (2012). Home care for older people in Sweden: A universal model in transition. Health Soc. Care Community.

[15] Swedish Association of Local Authorities and Regions (SKR) How to Handle the Work Environment Responsibility: The Elected Representatives Employer Role in Municipalities, County Councils and Regions. (Så Klarar du Arbetsmiljöansvaret: Förtroendevaldas Arbetsgivareroll i Kommuner, Landsting och Regioner). https://webbutik.skr.se/sv/artiklar/sa-klarar-du-arbetsmiljoansvaret.html.

[16] Swedish Work Environment Authority Organisational and Social Work Environment. AFS 2015:4. https://www.av.se/globalassets/filer/publikationer/bocker/books/the-organisational-and-social-work-environment-h457.pdf.

[17] The National Board of Health and Welfare (Socialstyrelsen). The Values in Social Service Care for the Elderly 2012:3. (Värdegrunden i socialtjänstensomsorg om äldre). https://www.socialstyrelsen.se/globalassets/sharepoint-dokument/artikelkatalog/foreskrifter-och-allmanna-rad/2012-2-20.pdf.

[18] Swedish Assosiation of Local Authority and Regions (SKR) Uppdrag och Samspel Mellan Ledande Politiker och Tjänstemän Assignments and Interaction Between Leading Politicians and Officials. https://webbutik.skr.se/bilder/artiklar/pdf/7164-812-9.pdf.

[19] Swedish Work Environment Authority (Arbetsmiljöverket) Supervision of Psychosocial Work Environment in Elderly Care. (Tillsyn av Psykosocial Arbetsmiljö Inom Äldreomsorgen). https://www.av.se/globalassets/filer/publikationer/rapporter/2018-007023_rapport_tillsyn_av_psykosocial_arbetsmiljo_inom_aldreomsorgen.pdf.

[20] Backhaus R., Verbeek H., van Rossum E., Capezuti E., Hamers J.P. (2015). Future distinguishing competencies of baccalaureate-educated registered nurses in nursing homes. Geriatr. Nurs..

[21] Gusi N., Adsuar J.C., Corzo H., del Pozo-Cruz B., Olivares P.R., Parraca J.A. (2012). Balance training reduces fear of falling and improves dynamic balance and isometric strength in institutionalised older people: A randomised trial. J. Physiother..

[22] Brett L., Noblet T., Jorgensen M., Georgiou A. (2019). The use of physiotherapy in nursing homes internationally: A systematic review. PLoS ONE.

[23] Andreassen M., Öhman A., Larsson Ranada Å. (2018). Assessing occupational performance in special housing in Sweden. Scand J. Occup. Ther..

[24] Elliott S., Leland N.E. (2018). Occupational Therapy Fall Prevention Interventions for Community-Dwelling Older Adults: A Systematic Review. Am. J. Occup. Ther..

[25] Bennett S., Laver K., Voigt-Radloff S., Letts L., Clemson L., Graff M., Wiseman J., Gitlin L. (2019). Occupational therapy for people with dementia and their family carers provided at home: A systematic review and meta-analysis. BMJ Open.

[26] The Governments Official Investigations (Statens Offentliga Utredningar-SOU) (2020). Elderly Care during the Pandemic. Interim Report by the Corona Commission. (Äldreomsorgen under Pandemin. Delbetänkande av Corona Kommissionen). http://www.sou.gov.se/wp-content/uploads/2020/12/SOU_2020_80_%C3%84ldreomsorgen-under-pandemin_webb.pdf.

[27] White E.M., Aiken L.H., Sloane D.M., McHugh M.D. (2020). Nursing home work environment, care quality, registered nurse burnout and job dissatisfaction. Geriatr. Nurs..

[28] Swedish Association of Local Authorities and Regions (SKR) (Sveriges Kommuner och Regioner-SKR). The Staff in the Welfare. (Personalen i Välfärden). https://skr.se/download/18.74ad29b6179e563a0cf8d88b/1624454995511/Personalen-i-valfarden.pdf.

[29] OECD (2012). Sick on the Job? Myths and Realities About Mental Health and Work.

[30] Cameron J., Sadlo G., Hart A., Walker C. (2016). Return-to-work support for employees with mental health problems: Identifying and responding to key challenges of sick leave. Br. J. Occup. Ther..

[31] Lee J.Y., Shin J.H. (2020). Why Do They Stay? Intention to stay among registered nurses in nursing homes. Int. J. Environ. Res. Public Health.

[32] Porter S., Lexén A. (2021). Swedish occupational therapists’ considerations for leaving their profession: Outcomes from a national survey. Scand. J. Occup. Ther..

[33] Backman A., Sjögren K., Lövheim H., Edvardsson D. (2018). Job strain in nursing homes—Exploring the impact of leadership. J. Clin. Nurs..

[34] Lindberg P., Vingard E. (2012). Indicators of healthy work environments—A systematic review. Work.

[35] The Governments Official Investigations (Statens offentliga utredningar-SOU 1 Read me! National Quality Plan for Care and Nursing for the Elderly Part 1. (Läs mig! Nationell Kvalitetsplan för vård och Omsorg om Äldre Personer del.). https://www.regeringen.se/4969b7/contentassets/9378aff4b35a427c99b772345af79539/sou-2017_21_webb_del1.pdf.

[36] Swedish Association of Local Authorities and Regions (SKR) Facts About Elderly Care in The Light of the Corona Pandemic. (Fakta om Äldreomsorgen i Ljuset av corona Pandemin). https://skr.se/download/18.7c4d4e89178e99232a38699/1618921734063/Fakta_om_aldreomsorgen_i_ljuset_av_coronapandemin.pdf.

[37] The Governments Official Investigations (Statens Offentliga Utredningar-SOU) Read me! National Quality Plan for Care And Nursing For The Elderly Part 2. (Läs mig! Nationell Kvalitetsplan för vård och Omsorg om ÄLDRE Personer Del 2). https://www.regeringen.se/4969b7/contentassets/9378aff4b35a427c99b772345af79539/sou-2017_21_webb_del2_hela.pdf.

[38] Skagert K., Dellve L., Eklöf M., Pousette A., Ahlborg G. (2008). Leaders’ strategies for dealing with own and their subordinates’ stress in public human service organisations. Appl. Ergon..

[39] The Health and Social Care Inspectorate (Inspektionen för vård och Omsorg IVO) What Have IVO Seen? 2020. (Vad har IVO sett?). https://www.ivo.se/globalassets/dokument/publicerat/rapporter/rapporter-2021/ivo_vhis-2020.pdf.

[40] Giorgi G., Lecca L.I., Alessio F., Finstad G.L., Bondanini G., Lulli L.G., Arcangeli G., Mucci N. (2020). COVID-19-Related Mental Health Effects in the Workplace: A Narrative Review. Int. J. Environ. Res. Public Health.

[41] Charmaz K. (2014). Constructing Grounded Theory.

[42] WHO (2001). World Medical Association Declaration of Helsinki—Ethical principles for medical research involving human subjects. Bull. World Health Organ..

[43] Dhaini S.R., Zúñiga F., Ausserhofer D., Simon M., Kunz R., De Geest S., Schwendimann R. (2016). Care workers health in Swiss nursing homes and its association with psychosocial work environment: A cross-sectional study. Int. J. Nurs. Stud..

[44] AFA Insurance (AFA Försökringar). Mental Health Diagnoses in the Contact Professions in Health Care, School and Care (Psykiska Diagnoser i Kontaktyrken Inom Vård, Skola och Omsorg). https://www.afaforsakring.se/globalassets/forebyggande/analys-och-statistik/f6345-psykiska-diagnoser.pdf.

[45] Gyllensten K., Wentz K., Håkansson C., Hagberg M., Nilsson K. (2019). Older assistant nurses’ motivation for a full or extended working life. Ageing Soc..

[46] Rundle C.W., Presley C.L., Militello M., Barber C., Powell D.L., Jacob S.E., Atwater A.R., Watsky K.L., Yu J., Dunnick C.A. (2020). Hand hygiene during COVID-19: Recommendations from the American Contact Dermatitis Society. J. Am. Acad. Dermatol..

[47] Falkenström E., Höglund A.T. (2019). “There is total silence here”. Ethical competence and interorganizational learing in healhtcarae goverance. J. Health Organ. Manag..

[48] Werntoft E., Edberg A.-K. (2015). Swedish politicans’ view of obstracles when dealing with priority settings in health care. J. Health Organ. Manag..

[49] Nilsson E., Nilsson K. (2017). Time for caring? Elderly care employees’ occupational activities in the cross draft between their work priorities,‘must-do’s’ and meaningfulness. Int. J. Care Coord..

